# Supplementary material for: The Impact of Dietary Counselling on Achieving or Maintaining Normal Nutritional Status in Patients with Early and Locally Advanced Breast Cancer Undergoing Perioperative Chemotherapy
Source: Nutrients. 2022 Jun 18;14(12):2541. doi: 10.3390/nu14122541 (PMC9228308; doi:10.3390/nu14122541)
Supplement: Supplementary file 1 [file nutrients-14-02541-s001.zip › nutrients-1750604-supplementary.pdf]

Questionnaire

I would like to ask you to complete the following questionnaire, which is designed to assess your diet and is part of the care of the dietetic clinic during perioperative chemotherapy. It is completely anonymous and the results will only be used for scientific purposes.

Thank you very much!

1) How many meals do you eat a day?

- ☐ **1**
- ☐ **2**
- ☐ **3**
- ☐ **4**
- ☐ **5**
- ☐ **>5**

2 Do you eat fried foods/meals?

- ☐ **Yes**
- ☐ **No**

3 How often do you eat vegetables?

- ☐ **Several times a day**
- ☐ **Once a day**
- ☐ **Several times a week**
- ☐ **Several times a month**
- ☐ **Not at all**

4 How often did you eat fruit?

- ☐ **Several times a day**
- ☐ **Once a day**
- ☐ **Several times a week**
- ☐ **Several times a month**
- ☐ **Not at all**

5 How often did you eat fish?

- ☐ **Several times a week**
- ☐ **Once a week**
- ☐ **Several times a month**
- ☐ **Once a month**
- ☐ **I do not eat fish**

6 How often do you eat sweets?

- ☐ **Several times a day**
- ☐ **Once a day**
- ☐ **Several times a week**
- ☐ **Several times a month**
- ☐ **Not at all**

7 What type of meat do you eat most often?

- ☐ **Poultry**
- ☐ **Pork**
- ☐ **Beef**
- ☐ **Horsemeat**
- ☐ **Venison**

8 What type of bread do you eat most often?

- ☐ **White**
- ☐ **Dark**
- ☐ **Mixed**

9 How much water do you drink during the day?

- ☐ **<0,5l**
- ☐ **0.5l-1l**
- ☐ **1l-1,5l**
- ☐ **1,5l-2l**
- ☐ **>2l**

10. How often do you consume alcoholic beverages?

- ☐ **Several times a day**
- ☐ **Once a day**
- ☐ **Several times a week**
- ☐ **Several times a month**
- ☐ **Not at all**

11 Do you use unconventional (natural) healing methods such as (Please tick all that apply):

- ☐ **Drinking beetroot juice**
- ☐ **Drinking tea made from cereal grass juice**
- ☐ **Drinking fennel seed tea and astragalus tea**
- ☐ **Eating saffron**
- ☐ **Eating sprouts**
- ☐ **Eating seaweed**
- ☐ **Eating linseed**
- ☐ **Eating apricot kernels**

- **Other. Which ones? ....**
- **No**

12 Are you taking statins?

- **Yes**
- **No**

Metrics

Age ....

Cancer:

o Poneopausal

o Premenopausal

Diabetes:

o Yes

o No
